# Effect of Liposomal *Protium heptaphyllum* (Alb.) March Extract in the Treatment of Obesity Induced by High-Calorie Diet

**DOI:** 10.3390/biology13070535

**Published:** 2024-07-17

**Authors:** Naiéle Sartori Patias, Eveline Aparecida Isquierdo Fonseca de Queiroz, Stela Regina Ferrarini, Gisele Facholi Bomfim, Danilo Henrique Aguiar, Adilson Paulo Sinhorin, Alexandre Aymberé Bello, Geovana Vicentini Fazolo da Silva, Larissa Cavalheiro, Valéria Dornelles Gindri Sinhorin

**Affiliations:** 1 Postgraduate Program in Biotechnology and Biodiversity of the Pro Centro-Oeste Network, Federal University of Mato Grosso, Sinop 78550-728, MT, Brazil; nai.sartori@gmail.com (N.S.P.); adilson.sinhorin@ufmt.br (A.P.S.); 2 NUPADS—Health Research and Teaching Support Center, Institute of Health Science, Federal University of Mato Grosso, Sinop 78550-728, MT, Brazil; eveline.queiroz@ufmt.br (E.A.I.F.d.Q.); gisele.bomfim@ufmt.br (G.F.B.); 3 Pharmaceutical Nanotechnology Laboratory, Postgraduate Program in Health Sciences, Federal University of Mato Grosso, Sinop 78550-728, MT, Brazil; stela.ferrarini@ufmt.br; 4 Institute of Natural, Human and Social Sciences, Federal University of Mato Grosso, Sinop 78550-728, MT, Brazil; dha.danilo@gmail.com (D.H.A.); larissacavalheiro@gmail.com (L.C.); 5 Institute of Health Science, Federal University of Mato Grosso, Sinop 78550-728, MT, Brazil; alexandre_aymbere@hotmail.com (A.A.B.); fazolog22@gmail.com (G.V.F.d.S.)

**Keywords:** obesity, *Protium heptaphyllum*, cytokines, oxidative stress, metabolism

## Abstract

**Simple Summary:**

Obesity is a chronic disease caused by excessive consumption of high-calorie foods that is associated with conditions such as dyslipidemia, diabetes, and cancer. *Protium heptaphyllum* (*P. heptaphyllum*) is known in folk medicine for its analgesic, anti-inflammatory, and healing properties, and its resin has demonstrated anti-obesity effects. This study aimed to evaluate the impact of liposomes containing the ethyl acetate fraction from *P. heptaphyllum* leaves on obesity in male Wistar rats. The animals became obese through a high-calorie diet and were treated with a liposome formulation containing *P. heptaphyllum* for 14 days. The study evaluated several parameters in adipose and liver tissues. Despite some positive effects on liver function and inflammation, the treatment did not attenuate global changes related to obesity, such as weight gain and fat accumulation. This highlights the complexity of obesity treatment and the varied responses of different organs to *P. heptaphyllum* liposomes.

**Abstract:**

Obesity, a chronic disease, resulted from excessive consumption of high-calorie foods, leading to an energy imbalance. *Protium heptaphyllum* (*P. heptaphyllum*) was used in folk medicine for its analgesic, anti-inflammatory, and healing properties. The association of the extract from *P. heptaphyllum* with nanotechnology was innovative for combining high technology with active ingredients that are easily accessible in the Amazon region. This study evaluated the effect of liposomes containing the ethyl acetate fraction of the crude extract of *P. heptaphyllum* leaves on obesity. Male Wistar rats treated with a high-calorie diet for 8 weeks to induce obesity received treatment with the liposome formulation containing *P. heptaphyllum* extract (1 mg/kg/day, via gavage) for 14 days. Morphological, metabolic, redox status, immunological, and histological parameters were evaluated in the adipose and liver tissue of the animals. The groups were divided as follows: C: control; P: liposomes containing extract; O: obese, and OP: obese + liposomes containing extract. The obesity model resulted in increases in body weight, caloric intake, body fat weight, and in the lipid profile. In adipose tissue, P decreased SOD (superoxide dismutase) activity in obese animals. In the liver, a positive modulation of the extract was observed in relation to glucose, amino acids, lactate, hepatoprotective action, and anti-inflammatory activity, with a decrease in interleukin 1β (IL-1β) in obese animals. The results showed an improvement in the functional and inflammatory aspects, but the treatment was not effective in alleviating general changes related to obesity, such as weight gain, fat, glucose, triglycerides, and inflammation in adipose tissue, highlighting the complexity of responses in different organs during obesity and treatment with *P. heptaphyllum*.

## 1. Introduction

Obesity is prevalent and growing, affecting all ages, and is considered a significant public health problem [1,2]. It results from energy imbalance, especially due to excessive consumption of high-calorie foods [3]. Furthermore, it is directly associated with metabolic syndrome, with oxidative stress as one of the main triggers of complications, including dyslipidemia, diabetes, hypertension, musculoskeletal disorders, and cancer [4,5]. The biochemical mechanisms involved include nicotinamide adenine dinucleotide phosphate (NAPDH) oxidase activation, oxidative phosphorylation, glyceraldehyde auto oxidation, protein kinase C activation, polyol and hexosamine pathways, hyperleptinemia, low antioxidant defense, postprandial reactive oxygen species generation, and chronic inflammation [6].

Chronic inflammation in adipose tissue produces pro-inflammatory chemokines and cytokines, stimulating immune cells from circulation to adipose tissue [7]. Obesity increases pro-inflammatory adipokines, such as interleukin-6 (IL-6), Alpha Tumor Necrosis Factor (TNF-α), adiposin, angiotensinogen, leptin, resistin, and visfatin, while decreasing the expression of anti-inflammatory adipokines, such as interleukin-10 (IL-10) and adiponectin, both in the systemic circulation and in tissues [8].

Herbal medicines offer an effective, affordable solution with less side effects to treat these disorders [9]. The use of these medications, which control oxidative stress and balance lipids, has been widely tested in therapeutic interventions [10]. However, more studies are needed to investigate the biological activity and toxicity of plants, aiming to attract investment from the pharmaceutical industry [9].

Nanotechnology has revolutionized the pharmaceutical sector, especially with advances in the use of liposomes (lipid vesicles), for more effective drug delivery [11]. Liposomes are vesicles formed by one or more phospholipid bilayers oriented concentrically around an aqueous compartment. Liposomes offer advantages over other nanosystems, such as their ability to encapsulate hydrophilic and lipophilic drugs depending on the affinity of the incorporated substance [12,13]. Liposomes can be produced on a nano- or micrometric scale, depending on the manufacturing technique [14]. Obtaining these vesicles incurs low manufacturing costs and is easily scalable compared to polymeric nanocapsules, for example. These promising liposomes contribute to safer and more effective medicines [15], especially when combined with plant extracts, expanding their therapeutic possibilities. This convergence between nanotechnology, liposomes, and plant extracts marks an evolution in pharmacology, facilitating the development of personalized and sustainable medicines [16,17]. This is because the encapsulation and delivery of flavonoids has resulted in the design of an innovative liposomal encapsulation technology to effectively deliver flavonoids to specific cellular targets and organelles [18].

Plant substances are promising sources for new medicines [19,20]. *Protium heptaphyllum* (*P. heptaphyllum*), known as “almacega” or “breu blanco”, is native to the Amazon [19,20,21] and is used in folk medicine for its stimulating, anti-inflammatory, and healing properties. Belonging to the Burseraceae family, with 18 genera and more than 700 species, the genus *Protium* is the main member [22]. Rich in triterpenes and flavonoids, *P. heptaphyllum* resin has demonstrated several bioactive properties, such as anti-inflammatory, antidepressant, anti-obesity, gastroprotective, and antibacterial actions [23,24,25,26,27]. Previous studies have investigated the effect of *P. heptaphyllum* leaf extracts against oxidative stress in mice, demonstrating antioxidant, hepatoprotective, and hypoglycemic properties [28].

Bioprospecting for natural products offers significant benefits to humanity, as biodiversity represents a valuable genetic library with future costs and benefits not yet fully known, with the Plantae kingdom being a crucial source of traditional medicine and an excellent reservoir for the discovery of new compounds with effective bioactives against difficult-to-treat diseases [29]. Thus, considering the anti-obesity effect already described by the resin and the antioxidant effect identified by the plant’s leaves, and knowing that there is an association between obesity and oxidative stress, this study aimed to demonstrate the therapeutic potential of the ethyl acetate fraction from the crude extract of *P. heptaphyllum* leaves, which are rich in flavonoids, in liposome form in an induced obesity model, according to a study by Patias et al. [28].

## 2. Material and Methods

### 2.1. Extract Preparation

The work was developed at the Integrated Chemical Sciences Research Laboratories (LIPEQ, Natural Products Chemistry Laboratory and Biochemistry Laboratory) at the Federal University of Mato Grosso, Campus of Sinop/UFMT. The exsicata is deposited in the collection of the Herbarium Centro-Norte-Mato-Grossense (CNMT) of the Federal University of Mato Grosso, Sinop campus, under number 625.

The leaves of *P. heptaphyllum* were selected and dried, crushed to powder, and then macerated with ethanol for seven days. Chlorophyll was removed with activated charcoal, then the extract was rotary-evaporated and lyophilized to be subjected to tests to identify functional groups. Part of the extract was fractionated in a silica gel chromatographic column with a gradient of solvents of increasing polarity, procedures that are more detailed in Reference [28]. After fractionation, the ethyl acetate fraction from *P. heptaphyllum* was chosen to be used in the development of liposomes, as it presented a greater amount of flavonoids.

#### 2.1.1. Development of Liposomes

Liposomes containing the active ingredient were developed using the reversed-phase evaporation method [30], followed by extrusion of the lipid film. First, soy phosphatidylcholine (sPC) and cholesterol (COL) were diluted in chloroform in a molar ratio of 4:1. The aqueous phase was prepared containing phosphate-buffered saline (pH 7.4) and polysorbate 80 and kept in ultrasound for 5 min. Subsequently, 1 mL of the aqueous phase was added to the organic phase and kept under ultrasound (5 min), and the organic solvent was removed by rotary evaporation at 25 °C until the formation of a thin film. This film was hydrated with the remainder of the aqueous phase and kept under stirring for 30 min. Finally, the liposome was subjected to a micelle extrusion process with 10 extrusion cycles using polycarbonate membranes with pores of decreasing size. The liposome containing the *P. heptaphyllum* extract will be called LP_EB,_ and the liposome without the active ingredient will be called LP_BR_.

#### 2.1.2. Physicochemical Characterization of Liposomes

The liposomes were characterized regarding their active content, the average diameter of the nanoparticles, polydispersity index (PDI), zeta potential, and pH. The formulations were stored at room temperature and protected from light for 30 days. The analyses were described with the mean and standard deviation of 3 batches.

#### 2.1.3. Particle Size Determination by Laser Diffractometry

The formulations were evaluated for mean equivalent sphere diameter (d4.3) and particle size distribution (Span) by laser diffraction using a Mastersizer^®^ 2000 (Malvern Instruments, Malvern, UK). The analyses, obtained at 25 °C from a small volume of the sample (enough to obscure the device between 2 and 8), were carried out in approximately 100 mL of distilled water as a dispersing medium.

#### 2.1.4. Particle Size Determination by Photon Correlation Spectroscopy

The average cumulant diameter (Z-average) and the polydispersity index (PDI), resulting from the application of the cumulant method, were measured at a detection angle of 173° (25 °C), by photon correlation spectroscopy analysis (Zetasizer^®^ NanoZS model ZEN 3600, Malvern Instruments, Malvern, UK) at 25 °C. The liposomes were diluted 500 times (*v*:*v*) in ultrapure water, filtered (0.45 µm membrane), and analyzed using the average of 3 replications.

#### 2.1.5. Analysis of pH and Zeta Potential

pH determination was carried out using a potentiometer (Denver^®^ Instrument VB-10, New York, NY, USA) previously calibrated with pH 4.0 and 7.0 standards directly in the formulations. The results represent the average of three determinations. The zeta potential was determined after diluting the liposomes 500 times in a 10 mM·L^−1^ NaCl solution, previously filtered through a 0.45 µm membrane, with measurements being carried out in triplicate, analyzing the sample for its electrophoretic mobility.

#### 2.1.6. Identification and Measurement of Quercetrin in the Liposome

Previous work by the group with the extract of *Protium heptaphyllum* (Alb. March) identified and quantified the flavonoids quercetin, quercetin-3-β-D-glucoside, myricetin, and phenolic compounds [28]. In the present work, we identified another flavonoid, quercetrin. The analysis of this flavonoid was carried out using high-performance liquid chromatography with UV detection (HPLC-UV, Perkin Elmer^®^, Waltham, MA, USA). The analytical study was carried out using high-performance liquid chromatography (HPLC) on a Perkin Elmer Series 200 chromatograph (Cotati, Sonoma, CA, USA), equipped with an ultraviolet detector. The system consisted of a LiChrosorb 100 RP-18 stainless steel column (5 µm, 250 × 4 mm) and a pre-column with LiChrosorb RP-18 stationary phase (5 µm). The mobile phase consisted of a mixture of acetonitrile: water (90:10 *v*/*v*), and the flow rate used was 0.7 mL/min.

The dosage of quercetrin contained in the liposome was carried out in triplicate, and the method was validated according to Harmonization of Technical Requirements of Registration of Pharmaceutical for Humane Use and Resolution No. 899 of the National Health Surveillance Agency. The methodology was validated in terms of specificity, linearity, precision, accuracy, limits of detection, and quantification.

### 2.2. In Vivo Tests

#### 2.2.1. Malone Test

Firstly, Malone’s Hippocratic test was performed to create a dose curve of the LP_EB_ (0.5, 1.0, 2.5, and 5 mg/kg, using three animals for each concentration) to analyze the possible acute toxicity of the doses and the lethal dose [31]. From there, a dose was selected to administer to the animals.

#### 2.2.2. Animals and Treatment

For the animal study, male Wistar rats were used. All steps to authorize work with animals were approved by the Guidelines of the Ethics Committee on the Use of Animals of the Federal University of Mato Grosso no. 23108.031684/2021-21. Animals were used in numbers of eight per group over a 14-day treatment period.

Control animals were fed standard rodent chow (NUVILAB CR-1, Nuvital^®^, Colombo, Paraná, Brazil), and obese animals received hypercaloric chow (rich in lipids; 24.5% of energy coming from lipids) and water with sucrose (300 g/L). The model and composition of the diet were prepared according to References [32,33]. The high-calorie diet was developed in the laboratory and contained commercial food (NUVILAB CR-1), condensed milk, cornstarch biscuits, casein, lard, vitamins, and minerals (Table 1). The ingredients were first ground and then mixed with vitamins and minerals. The mixture was transformed into pellets, dried in a drying oven at 45 ± 5 °C for 48 h, turned after 24 h, and stored at −8 °C.

Throughout the experimental period, the animals were kept in polypropylene boxes, placed in an environment with a controlled temperature of 22 ± 2 °C and a 12 h light–dark cycle, with free access to water and food. The animals were distributed into 4 experimental groups according to whether they were treated with LP_EB_. We identified the groups as follows:

Group C: Control group;
Group P: Control group treated with LP_EB_;
Group O: Obese group
Group OP: Obese group treated with LP_EB_.

The acclimatization period was two weeks. The groups treated with LP_EB_ (P, OP) received the treatment dose defined by the Malone test [31], which was 1.0 mg/kg via gavage, once a day for 14 days. The control groups (C and O) received vehicle (water—1 mL/kg) via gavage also during the 14 days.

After the treatment period, the animals were fasted for 8 h. Blood was collected by cardiac puncture under anesthesia with Ketamine 50 mg/kg and Xilaxin 2 mg/kg, then the animals were euthanized by decapitation. Liver and adipose tissue samples were collected by dissection, washed with isotonic saline, and weighed to determine absolute (g) and relative weight (g/100 g of body weight). Furthermore, the weight of the mice (g) was evaluated, and tissue samples were stored frozen at −80 °C for later analysis, except for tissue samples collected for histological analyses, which were separated and processed immediately. Plasma was obtained from whole blood by centrifugation.

#### 2.2.3. Characterization of Obesity and Analysis of the Metabolic Parameters

To characterize and confirm the effectiveness of the model for inducing obesity using a high-calorie diet, obesity was assessed by determining the Adiposity Index = [(periepididymal fat + retroperitoneal fat)/body weight × 100]; the relative weight of periepididymal and retroperitoneal fats, and lean mass (soleus and EDL muscles) in grams/100 g of body weight.

Weight gain and water and feed intake were assessed from the 1st to the 8th week.

#### 2.2.4. Feed and Water Consumption

To evaluate daily food consumption, the rats were housed in polypropylene boxes and provided with free access to water and food. Initially, 500 g of food was placed in each box. After a period of 48 or 72 h (on weekends), the remaining amount of food was weighed. The difference obtained represented the volume consumed by the rats in the box, which was then divided by the number of animals present and the interval of 2 or 3 days (corresponding to the period between weighing). This procedure was repeated throughout the experiment, allowing for the evaluation of feed consumption from the beginning to the end of the treatment. The results were expressed in grams. The protocol used to analyze water consumption followed the same procedure adopted for feed consumption, considering that the amount of water supplied to the animals every two or three days was 1000 mL. The animals’ food consumption expressed in calories was obtained using a mathematical formula: group C and P: (amount consumed in food/day/rat (g) × 3.77 (kcal)) + (amount consumed in water/day/rat (mL) × 0 (kcal)) = value consumed in calories per day per rat (kcal); group O and OP: (amount consumed in food/day/rat (g) × 5.14 (kcal)) + (amount consumed in water/day/rat (mL) × 1.2 (kcal)) = amount consumed in calories per day per rat (kcal). The caloric value (kcal) of 1 g of feed was 3.77 kcal, that of 1 g of high-fat feed was 5.25 kcal, and 1.20 kcal for 1 mL of sugar-water (sucrose) solution [33].

#### 2.2.5. Intraperitoneal Insulin Tolerance Test (IPITT) and Oral Glucose Tolerance Test (OGTT)

For the Intraperitoneal Insulin Tolerance Test (IPITT), caudal blood samples were collected before and at 4, 8, 12, 16, and 20 min after an intraperitoneally injected regular insulin overload. The constant rate of disappearance of blood glucose during the test (KITT) was calculated based on linear regression of the Neperian logarithm of glucose concentration. The animals were evaluated on the tenth day of treatment.

In the Oral Glucose Tolerance Test (OGTT), a caudal blood sample was collected and corresponded to basal glycemia (T0), after a period of 15 h of food restriction. Next, glucose was administered at a dose of 2.5 g/kg of body weight via gavage (0.5 g/mL glucose solution). Afterward, blood samples were collected at 15, 30, 60, 90, and 120 min after glucose administration corresponding to T15′, T30′, T60′, T90′, and T120′. Blood glucose was determined using a glucometer and strips (SENS II^®^ Glucometer, Injex, Brazil). The result was calculated using the value of the area under the curve, represented by glucose [(mg/dL × min^−1^) × 1000].

#### 2.2.6. Biochemical Analyses in Liver, Adipose Tissue, and Plasma

The dosages of enzymatic antioxidant activity of CAT (catalase) were determined according to Reference [34], SOD (superoxide dismutase) according to Reference [35], GST (glutathione-S-transferase) according to Reference [36], and GPx (glutathione peroxidase) was measured according to Reference [37]. The dosage of the non-enzymatic antioxidant GSH (reduced glutathione) was carried out according to Reference [38], and that of ascorbic acid (AAS) was measured according to Reference [39]. The indirect markers of oxidative damage evaluated were TBARS (substances reactive to thiobarbituric acid) and carbonyl (carbonylated proteins) according to the technique described by References [40,41], respectively. The protein content of the tissues, except for the ascorbic acid dosage, was determined according to Reference [42].

The biochemical parameters (glucose, aspartate aminotransferase—AST, alanine aminotransaminase—ALT, alkaline phosphatase—ALP, amylase, lipase, creatinine, lactate and total proteins) and the lipid profile (total cholesterol, triglycerides, LDL—low-density lipoprotein, HDL—high-density lipoprotein) from blood plasma were analyzed at a partner Clinical Analysis Laboratory using a biochemical analyzer (XT-18000 Sysmex, Roche, Hitachi Ltd., Tokyo, Japan).

Glucose was determined according to Reference [43], glycogen according to Reference [44], and lactate was estimated according to Reference [45]. The protein content was determined by the method described in Reference [42], the amino acid dosage was carried out according to Reference [46], and ammonia was measured according to the reference method in [47].

#### 2.2.7. Histological Analyses of Liver Tissue

For histological analyses of the liver, samples of liver tissue were fixed in buffered formalin and dehydrated in various concentrations of ethanol, then immersed in resin, sectioned into 3 µm transverse sections, and stained with hematoxylin and eosin (H&E), visualized under an optical microscope, and photographed as described by a previous study [48]. Liver structures were evaluated for hepatic inflammation, steatosis, hepatocyte degeneration, and fibrosis.

#### 2.2.8. Immunological Analysis by ELISA

The homogenate obtained by diluting and homogenizing 50 mg of each tissue in phosphate-buffered saline (PBS) was centrifuged, and the supernatant was separated for use in measuring cytokines. The cytokines IL-6, IL-10, TNF-α, interferon gamma (IFN-γ), interleukin 17 (IL-17), and interleukin 1β (IL-1β) were measured according to commercial kits from R&D System, by sandwich ELISA technique. The concentrations of each cytokine were calculated based on the linear regression equation of the standard curve obtained with recombinant rat cytokines.

### 2.3. Data Analysis

Initially, the data passed the Kolmogorov–Smirnov normality test. The ANOVA (two-way) analysis of variance test was performed followed by the Tukey–Kramer multiple comparison test for comparing more than two means. Furthermore, in cases where the results did not pass the normality test, the Kruskal–Wallis’s test and Dunn’s post-test were performed. The minimum acceptable significance level was *p* ˂ 0.05. The results were expressed as mean +/− standard deviation and/or median and total range. The graphs were generated and the results analyzed using the Graph Pad Prism 8.0 statistical program.

## 3. Results

### 3.1. Development and Characterization of Liposomes

The liposomes resulted in formulations with a homogeneous macroscopic, milky, opalescent, and slightly greenish appearance, a characteristic expected with the presence of *P. heptaphyllum* extract (LP_EP_), and the formulations without the extract presented a whitish color (LP_BR_). Table 2 presents the values referring to the average diameter, span, pH, and zeta potential obtained for the average analyses and their respective standard deviation of the micelles developed.

The average diameter of the vesicles of the LP_BR_ and LP_EP_ formulations, by laser diffraction, was below 300 nm, presenting a monomodal system, without the presence of nanometric-sized microparticles. Polydispersity demonstrated low variation, with SPAN values around 2.0. The average size of the vesicles by photon correlation spectroscopy corroborates the nanometric diameter already found by laser diffraction. The polydispersity indices (PDI) were close to 0.210, indicating low polydispersity, which makes the colloidal system stable. Zeta potential (ζ) analyses demonstrated values around 20 mv, in modulus, indicating the steric stabilization method, as expected for these nanosystems.

The non-ionic surfactants in the formulations perform steric stabilization, causing a reduction in zeta potential [49]. The pH values of the formulations were close to neutrality, an expected result since a buffer solution (pH 7.4) was used as a vehicle, in addition to the cationic group in the formulation, resulting from phosphatidylcholine, which also provided a neutral pH to the liposomes [50,51]. The analytical method developed in HPLC-UV was validated and shown to be linear, precise, accurate, and robust. The content of the quercetrin compound present in the extract was 98.8 ± 0.9%, with a retention time of 34.0 min.

### 3.2. Food Consumption of Animals from the First to Eighth Week and Tissue Weight

A significant difference was observed in the final weight of the animals submitted to the high-calorie diet, and a significant increase was seen in the average weight gain of O and OP groups compared to the C group. The statistical results revealed disparities in food intake between the groups submitted to the hypercaloric diet (O and OP), with a notable reduction in feed intake and a corresponding increase in water intake in these groups. Additionally, a progressive increase in caloric intake was observed throughout the study period in the groups submitted to a high-calorie diet, and treatment with the *P. heptaphyllum* liposome did not reverse this parameter (Table 3).

Regarding tissue weight, there was an increase in epididymal and retroperitoneal adipose tissue in O and OP groups compared to the C group. Treatment with the liposome increased this same adipose tissue when comparing obese animals. There was an increase in liver weight in animals belonging to these same groups (O and OP group compared to C group), but the hepatosomatic index did not change in the groups analyzed (Table 3).

**Figure 1:**
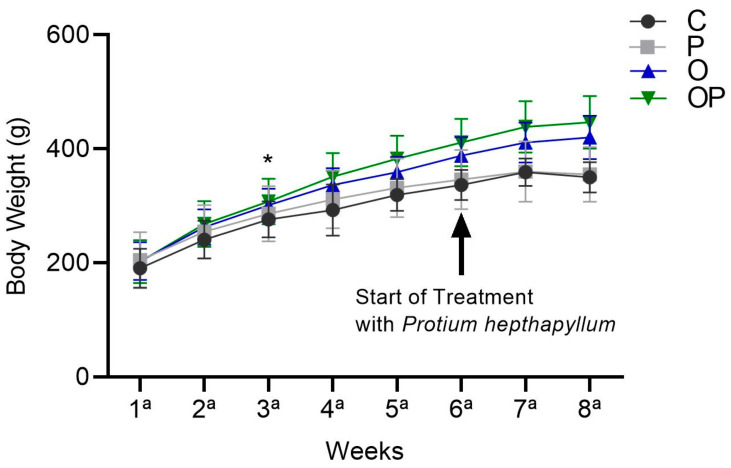
Weight evolution of animals in the Control (C), Protium (P), Obese (O), and Obese Protium (OP) groups between the 1st and 8th week of the experimental protocol (*n* = 8 animals per group). Results are presented as mean ± standard deviation. ANOVA (two-way) followed by Tukey’s post-hoc test. * *p* < 0.05 vs. C.

There was an increase in the average body weight of the O and OP groups from the sixth week until the end of treatment in the eighth week, and the *P. heptaphyllum* liposome was unable to reduce this increase (Figure 1).

**Figure 2:**
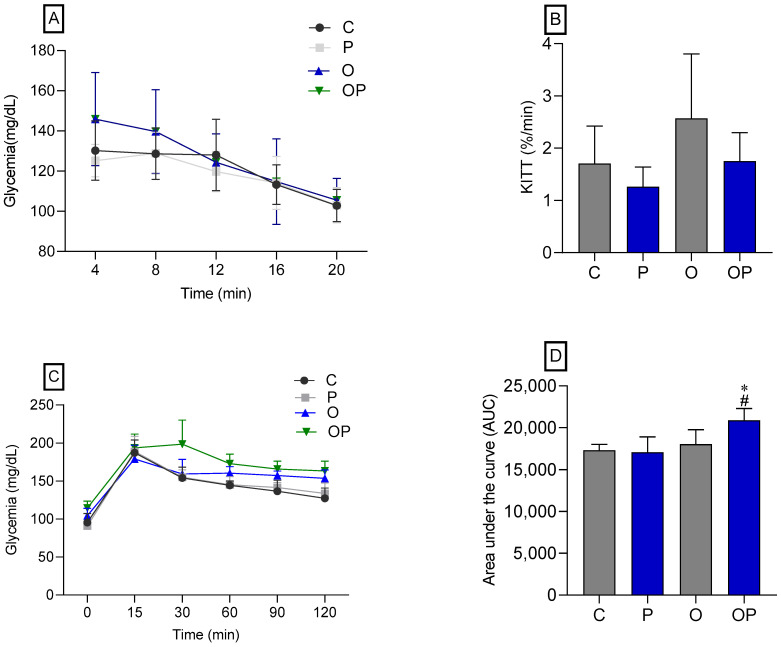
Glycemic curve (**A**) and glucose decay constant (KITT) (**B)**, both obtained through the Intraperitoneal Insulin Tolerance Test (IPITT) of the Control (C), Protium (P), Obese (O), and Obese Protium (OP) groups. Glycemic curve (**C**) and area under the curve (AUC) (**D**), obtained through the Oral Glucose Tolerance Test (OGTT) of the Control (C), Protium (P), Obese (O), and Obese Protium (OP) groups (*n* = 8 per experimental group). Results are presented as mean ± standard deviation. ANOVA (two-way) followed by Tukey’s post-hoc test. * *p* < 0.05 vs. C; # *p* < 0.05 vs. O.

There was no statistical difference between the groups concerning IPITT (Figure 2A,B), demonstrating that there was no change in insulin sensitivity in animals treated or not treated with *P. heptaphylllum*. Regarding the OGTT, no statistical differences were observed in the glycemic curve (Figure 2C), but we found that the OP group showed greater glucose intolerance, which was evidenced by the area under the curve (Figure 2D) when compared to the C and O groups.

### 3.3. Analysis of Plasma Biochemical Parameters

The high-calorie diet increased glucose levels in the O and OP groups when compared to the C group (Table 4), and the *P. heptaphyllum* extract was not able to reverse the animals’ blood glucose levels within normal limits compared to reference values. To evaluate liver functions, serum activities of the enzymes alanine aminotransferase (ALT), aspartate aminotransferase (AST), and alkaline phosphatase (ALP) were measured. The enzymes ALT, AST, and ALP decreased significantly in the O and OP groups compared to the C group, and the P group also showed a decrease in the AST activity when compared to the C group (Table 4). Furthermore, for creatinine, there was a reduction in the P, O, and OP groups when compared to group C. On the other hand, the parameters of total proteins, amylase, and lipase did not demonstrate statistical differences between the groups studied, as detailed in Table 4.

Among the parameters related to the lipid profile, we observed a reduction in LDL cholesterol levels in the P and OP groups compared to the C group, as well as a reduction in VLDL levels in the P, O, and OP groups compared to the C group. However, for triglycerides, there was an increase in the OP group compared to the O group (Table 5). The TG/HDL ratio was increased in the OP group compared to the C and O groups. Regarding total cholesterol and HDL cholesterol, no significant changes attributed to the extract were observed in this 14-day experimental protocol.

### 3.4. Adipose Tissue

#### 3.4.1. Analysis of Parameters of Oxidative and Metabolic Stress in Adipose Tissue

A reduction in SOD enzyme activity was observed in the OP group compared to the O group. There were no significant changes between groups for CAT, GST, GSH, and ASA. On the other hand, TBARS levels increased in the O and OP groups compared to the C group, and carbonyl levels were reduced in the P group compared to the C group (Table 6).

Regarding metabolites, both glucose and total proteins did not reveal statistically significant differences between the groups. However, there was a reduction in glycogen levels in the P, O, and OP groups compared to the C group. Amino acids were reduced in the OP group compared to the C group. There was an increase in ammonia levels in groups P and O compared to group C. On the other hand, there was a reduction in these levels in the OP group when compared to the O group. As for lactate, an increase in levels was observed in the P group compared to the C group (Table 6).

#### 3.4.2. Analysis of Cytokines in Adipose Tissue

**Figure 3:**
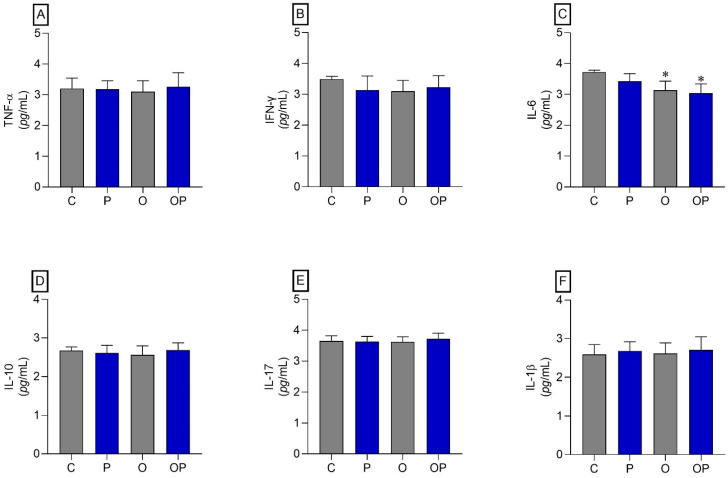
Adipose tissue cytokines. (**A**) TNF-α: Alpha Tumor Necrosis Factor, (**B**) IFN-γ: interferon gamma, (**C**) IL-6: interleukin 6 cytokines, (**D**) IL-10: interleukin 10, (**E**) IL-17: interleukin 17, and (**F**) IL-1β: interleukin 1β; *n* = 8 per experimental group. Value represents mean ± standard deviation. * *p* < 0.05 vs. C. For statistical analysis, data were log-transformed.

No statistically significant differences were identified between the groups in TNF-α, IFN-γ, IL-10, IL-17, and IL-1β (Figure 3A,B,D–F, respectively). However, regarding IL-6, a reduction in this cytokine was found in O and OP tissues when compared to group C (Figure 3C).

### 3.5. Liver Tissue

#### 3.5.1. Analysis of Parameters of Oxidative and Metabolic Stress in Liver Tissue

No changes were observed in the groups analyzed concerning the parameters of SOD, CAT, GST, GPx, GSH, ASA, and carbonyl; however, there was an increase in TBARS in the P and O groups when compared to the C group (Table 7).

Considering the levels of metabolites in the liver tissue, there was an increase in glucose concentration in the P, O, and OP groups compared to the C group. However, *P. heptaphyllum* was able to correct the levels in the OP group when compared to the O group. Glycogen concentration showed an increase in the P group compared to the C group. For amino acid levels, there was a decrease in the P and O groups compared to the C group, and *P. heptaphyllum* corrected these amino acid levels in the OP group when compared to the O group. The ammonia concentration showed a decrease in group P compared to group C, and lactate levels showed a decrease in group O compared to the group C; animals treated with extract liposomes (OP) recovered these values to control levels. Total proteins showed no statistical difference between the groups studied.

#### 3.5.2. Analysis of Cytokines in Liver Tissue

**Figure 4:**
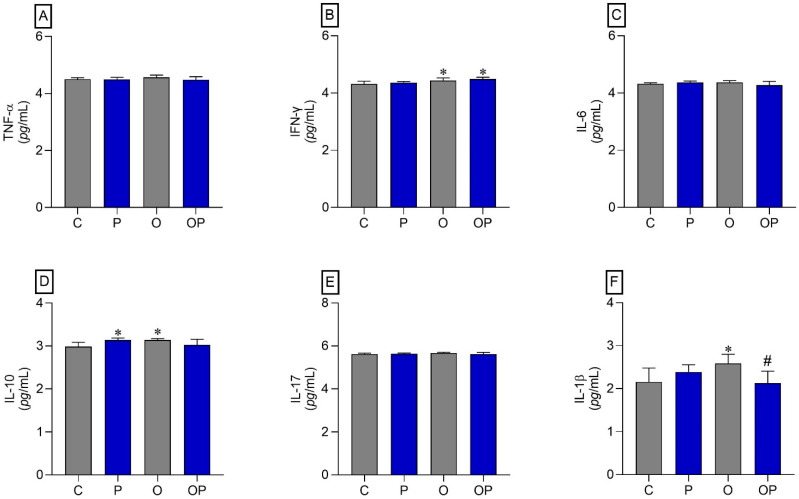
Liver tissue cytokines. (**A**) TNF-α: Alpha Tumor Necrosis Factor, (**B**) IFN-γ: interferon gamma, (**C**) IL-6: interleukin 6, (**D**) IL-10: interleukin 10, (**E**) IL-17: interleukin 17, and (**F**) IL-1β: interleukin 1β; *n* = 8 per experimental group. Value represents mean ± standard deviation. * *p* < 0.05 vs. C; # *p* < 0.05 vs. O. For statistical analysis, data were log transformed.

The results indicated that there was an increase in IFN-γ in the O and OP groups compared to the C group (Figure 4B). The cytokine IL-10 also showed an increase in the P and O groups when compared to the C group (Figure 4D). On the other hand, the cytokine IL-1β increased in group O compared to group C, and plant liposomes were effective in reducing the levels of this cytokine in group OP (Figure 4F). The cytokines TNF-α, IL-6, and IL-17 did not show statistically significant differences (Figure 4A,C,E, respectively).

#### 3.5.3. Histopathological Analysis of Liver Tissue

**Figure 5:**
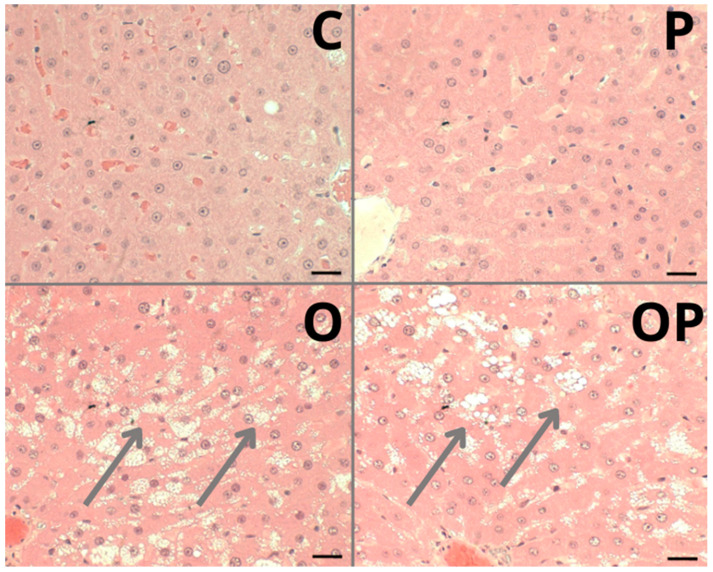
Photomicrograph of a sample from an individual from each liver tissue group showing different morphological changes in the Control (C), Protium (P), Obese (O) and Obese Protium (OP) groups. Steatosis: (arrows) groups O and OP. Resolution: Bar 30 μm.

The liver of animals fed a standard diet (C and P) showed well-formed nucleated hepatocytes, well-formed sinusoidal capillaries between hepatocyte plates, absence of inflammatory infiltrate, fibrous tissue, or lipid accumulation, and intact architecture compatible with a normal liver (Figure 5—C and P). The groups that received a high-calorie diet (O) and were treated with *P. heptaphyllum* liposomes (OP) showed hepatic steatosis with vesicular-appearing cytoplasm, with many fat droplets of various sizes scattered around (Figure 5—O and OP).

## 4. Discussion

We used nanotechnology to create liposomes from *P. heptaphyllum* extract, with the aim of making the product more effective and reducing possible adverse effects [13]. This approach improves the absorption of bioactive substances, such as flavonoids present in the extract, especially when administered orally [51]. The use of nanocarriers has become a common approach to reduce toxic effects and enhance active ingredient activity [52]. Liposomes were prepared by the method of lipid film hydration followed by extrusion [30]. This method is widely used due to its reproducibility and easy handling, resulting in an opalescent liquid that can be used directly after preparation. The liposomes showed a bluish white color due to the Tyndall effect, characteristic of concentrated colloidal solutions. LP_EP_ had an opalescent milky green color, with a slight odor characteristic of the extract used. All formulations were macroscopically homogeneous with a bluish reflection, resulting from the Brownian movement of the nanomicelles. The techniques used to evaluate the diameters of the formulations showed the presence of nanometric micelles, without any microscopic samples. Furthermore, the low polydispersity values demonstrated narrow size distributions and uniformity in mean diameters for all formulations developed. The pH neutrality is consistent with the composition of the nanosystems in this study. All these parameters qualified the LP_EP_ and LP_BR_ formulations for application in biological models.

The induced obesity model used in the present study was confirmed by the increase in average body weight and the accumulation of body fat, reflected in the increase in organ weight. Furthermore, comprehensive data such as adipose tissue accumulation and hyperglycemia, typical features of obesity and associated metabolic disorders, corroborate the effectiveness of the model. These findings are consistent with other studies that used obesity induction. For example, previous studies reported the induction of obesity in rats on a high-fat diet [53]. Similarly, a high-fat and high-protein diet, commonly used for weight loss, induced obesity in rats [54], and another study used an obesity induction protocol and treated rats with botryosphaeran [(1→3) (1→6)-β-d-glucan], obtaining beneficial metabolic, antioxidant, and anti-inflammatory effects [55]. Finally, it is claimed that high-fat diets are effective in modeling the metabolic disorders of human obesity in rodents [56]. Although the study protocol [54] is like ours, we were unable to observe positive changes in liposomes in the epididymal and retroperitoneal adipose tissue depots, since the results were greater in the OP group compared to the O group. This unexpected result may be attributed to the insufficient dose of the extract and the 14-day treatment period. The liposomal form of the extract may also have interfered with the bioavailability of the bioactive compounds. Studies show that obesity is associated with oxidative stress, and treatments with natural extracts can be toxic and should be used with caution, as they can cause adverse effects in the long term or at incorrect doses. Furthermore, the complexity of the herbal composition makes it difficult to determine the mechanisms of action, as discussed by studies that provide an overview of the scientific evidence on the use of herbal medicines in the treatment of obesity [57].

Animals that received a high-calorie diet (O) and were treated with liposomes (OP) showed a significantly larger area under the curve in the OGTT, suggesting signs of glucose intolerance. Hypercaloric diets induce metabolic disorders like human metabolic syndrome [56,58] and, because of obesity triggered by the high caloric value of the diet, changes in glycemia [59]. However, in this study, the obese groups (O and OP) presented high plasma glycemia, without reversal by treatment with *P. heptaphyllum,* but previous studies reveal the action of the resin of *P. heptaphyllum* in reducing plasma glucose in the face of obesity induced by a high-fat diet in rats [60]. Obesity is associated with increased liver enzymes, markers of liver function, with hepatic steatosis being common in obese individuals, which can cause liver damage and result in the release of these enzymes into the bloodstream [61,62]. The reduction observed in the activities of the hepatic enzymes ALT, AST, and ALP in the obese groups (O and OP) demonstrates that the model developed did not cause damage to the point of increasing these activities, nor did treatment with liposomes containing *P. heptaphyllum* interfere with these parameters. Previous studies showed that *P. heptaphyllum* resin attenuated the acute paracetamol-induced increase in serum ALT and AST activities in mice [19]. Accordingly, the activity of ALT, AST, and ALP enzymes increased in the plasma of mice exposed to paracetamol, but ethyl acetate extract of *P. heptaphyllum* decreased these activities by controlling ALT and AST activities [28]. Studies in rats on a high-fat diet for 15 weeks showed that *P. heptaphyllum* also triggered a decrease in liver enzyme activities [62]. Furthermore, we observed that the treated groups also showed a decrease in creatinine levels, in line with similar results in rats on a high-calorie diet and lycopene treatment [63]. We can observe that previous studies showed good results with *P. heptaphyllum*, and our results, despite not showing many advances in these parameters, may have been influenced by the treatment time and the dose of the extract. Added to that, the innovative methodology of liposomes presents challenges in understanding the bioactives involved.

Regarding the lipid profile, the P and OP groups showed a decrease in LDL and VLDL levels, which can be attributed to the flavonoids present in *P. heptaphyllum*, such as quercetin-3-β-D-glucoside, myricetin, and quercetrin. Studies have discussed the antioxidant potential and other biological activities of *P. heptaphyllum*, highlighting these bioactive compounds [64]. Furthermore, they demonstrated that quercetrin can reduce cholesterol and prevent atherosclerosis [65], reporting the lipid-lowering effects of the hydroalcoholic extract of *Solidago chilensis* and its main isolated constituent in cholesterol-fed rats, demonstrating the ability of quercetrin to reduce cholesterol and prevent atherosclerosis.

In addition, the triterpenes α- and β-amyrin present in *P. heptaphyllum* resin also reduce LDL and VLDL lipoproteins, such as in a study that investigated the antihyperglycemic and lipid-lowering effects of the mixture of triterpenes α- and β-amyrin from *P. heptaphyllum* in mice, showing a reduction in LDL and VLDL lipoproteins [66]. Another study investigated the bioactive triterpenes from *P. heptaphyllum* resin extract, noting their cholesterol-lowering potential, especially in LDL lipoproteins [67]. The hypercaloric diet applied was rich in lipids and not in carbohydrates, which explains the low production of hepatic TG and reduction in VLDL and LDL. However, an increase in triglycerides and the TG/HDL ratio was observed in the treated group (OP), associated with an increase in adipose tissue, crucial for triglyceride storage, since the fate of excess fat absorbed from the diet is delivered directly to adipose tissue for storage via chylomicrons [68]. Research has reported increased visceral and subcutaneous adipose tissue in children with acute pancreatitis, highlighting the importance of adipose tissue in triglyceride storage [69]. In addition, the relationship between the triglycerides/HDL index (TG/HDL) as a risk marker for metabolic syndrome and cardiovascular diseases was discussed, correlating it with the increase in adipose tissue and lipid profile [70]. The presence of the plant during this treatment period did not affect this marker. This link between high-fat diets and metabolic changes highlights the potential adverse effects of these diets on plasma and liver lipid profiles. Research has examined the effects of high-calorie diets on glucose homeostasis in rats, highlighting the influence of saturated and monounsaturated dietary lipids on plasma and liver lipid profiles [68].

Adipose tissue, a vital component of the human body, plays a fundamental role in metabolic and homeostatic functions. In the present study, an increase in TBARS levels in the adipose tissue of obese groups (O and OP) was observed, indicating greater oxidative stress and possible cellular damage in this tissue. On the other hand, other studies have already demonstrated positive effects of *P. heptaphyllum* in reducing TBARS and carbonyl. For instance, the investigation of the anti-inflammatory effect of the triterpenes α- and β-amyrin from *P. heptaphyllum* in a model of acute periodontitis in rats showed a significant reduction in oxidative stress, especially TBARS [23]. Another study [28] observed carbonyl reduction in kidney tissue samples from mice exposed to paracetamol and treated with the ethyl acetate fraction of *P. heptaphyllum*. On the other hand, the activity of the SOD enzyme decreased in the OP group when compared to the O group. Several studies demonstrate the antioxidant potential of flavonoids, especially quercetin, which acts directly on antioxidant enzymes. In this context, the antioxidant capacities of flavonoids present in cherries (*Prunus pseudocerasus*) were evaluated, including astragalin, cyanidin-3-O-glucoside, cinaroside, quercetin, rutin, and vitexin, and treatments with high doses of cyanidin-3-O-glucoside and rutin increased SOD activities in the serum, liver, kidney, and heart of mice, while reducing the level of MDA (malondialdehyde) in these tissues [71]. Quercetin, a bioactive flavonoid with several antioxidant properties, has been highlighted for its biological importance and its role in protecting against oxidative stress [72]. Additionally, studies analyzing the antioxidant activities of quercetin and its complexes have observed significant beneficial effects for medicinal applications, particularly in reducing oxidative damage [73]. However, in this study, we did not observe a pattern of protection of liposomes containing the extract in this tissue against enzymatic and non-enzymatic activities or inflammatory markers, even after a 14-day treatment. It is possible that a longer treatment time will be required.

This research is pioneering with *P. heptaphyllum* extract liposomes, including redox and metabolic analyses, demonstrating an innovative approach. The reduction in glycogen in groups O and OP in adipose tissue suggests changes in energy metabolism associated with insulin resistance in obesity, indicating a possible reduction in glucose storage capacity [74]. The decrease in amino acids and ammonia in the OP group reflects specific metabolic adjustments, indicating a positive response to treatment [75]. Obesity can affect amino acid metabolism, resulting in changes in the production and excretion of ammonia, a metabolic byproduct whose obesity-related imbalances can lead to elevated levels of this compound [76].

When natural liver protective mechanisms fail, liver damage can occur. Thus, treatment with liposomes for 14 days in obese animals did not promote changes in enzymatic antioxidants (SOD, CAT, GST, and GPx), but the TBARS marker, a sign of lipid damage, remained elevated (groups P and O) in liver tissue, without causing protection against obesity, and both triggered lipoperoxidation during this treatment period. Previous studies from our group highlighted the ability of *P. heptaphyllum* extracts to reduce hepatic oxidative stress in a paracetamol-induced liver damage model [28]. It is known that quercitrin present in *P. heptaphyllum* extract is recognized for its bioactivity, and it has been studied in various health conditions, exhibiting antioxidant, anti-inflammatory, and antimicrobial properties [77]. Furthermore, some authors [78] have suggested that flavonoids, including quercetin, directly impact mitochondrial processes, indicating potential to counteract complications associated with obesity. However, even though the liposomes under study contain these flavonoids, it is important to explain that we carried out a 14-day treatment, with a different form of dispensing, and it is possible that these conditions were not ideal for the possible benefits in this organ under obesity induction.

In the obese group treated with *P. heptaphyllum* (OP), there was a reduction in glucose levels compared to the obese group, indicating a possible benefit of the treatment on liver tissue. *P. heptaphyllum* restored amino acid and lactate levels in the OP group, suggesting modulation of these parameters attributable to flavonoids such as quercetrin present in the plant. Studies [79] have indicated that high concentrations of plasma glucose and lactate result in greater uptake in peripheral tissues, regardless of the type of diet. Furthermore, the liposome per se promoted an increase in glucose and hepatic glycogen stores combined with a reduction in amino acids and ammonia. The liver is responsible for carrying out the urea cycle, an important route for ammonia excretion. It is possible that the amino acids provided their carbon skeletons to produce glycogen, and the ammonia generated was efficiently destined for its elimination via the urea cycle [70].

Flavonoids such as quercetin are recognized for anti-inflammatory and cytokine immunomodulatory properties. In the liver tissue, there was an increase in IFN-γ (groups O and OP), IL-10 (groups P and O), and IL-1β (group O) compared to the control, showing an inflammatory process mediated by T helper lymphocytes 1 (Th1) and inflammasome activation in the obese group. The increase in IL-10 in obese individuals may be due to an attempt to control the inflammatory process in this tissue. The use of liposomes in obese animals managed to reduce IL-1β levels in the liver, probably reducing the NLRP3 (NOD-like receptor family pyrin domain-containing 3) and inflammasome activation pathway. The anti-inflammatory effect related to *P. heptaphyllum* was reported in an animal model of periodontitis, where the plant isolates, α- and β-amyrin, were administered 2 h before the induction of periodontitis. After 6 h, there was a decrease in TNF-α levels in treated animals, although other cytokines were not measured [23]. Previous studies [80] also highlighted the ability to reduce inflammatory cytokines in inflammation models where the effects of quercetin-loaded liposomes were investigated in a mouse model of sepsis, demonstrating that liposomal encapsulation promoted the inhibitory effects of quercetin on lung-mediated inflammation by macrophages, reducing mortality without apparent toxicity.

Histological evaluation revealed hepatic steatosis in groups O and OP. Unfortunately, treatment with liposomes failed to prevent this change caused by obesity, contradicting the findings of other studies that also investigated the same plant. However, using other components present in it, such as the triterpenes α- and β-amyrin extracted from the trunk resin, conferred significant protection against acetaminophen-induced liver injury in mice, preventing hepatic congestion and centrilobular necrosis [19]. In addition, other works with *P. heptaphyllum* resin showed that it prevents microgoticular steatosis and liver inflammation in mice fed a high-fat diet, preserving normal liver morphology [60], and administration of α- and β-amyrin in streptozotocin-treated diabetic mice protects the islets of Langerhans from cellular destruction, maintaining the morphological integrity of the pancreas, like normal controls [66]. Additionally, studies with the same resins from *P. heptaphyllum* showed prevention of inflammatory infiltrations and the accumulation of lipid droplets in the liver of mice fed a high-fat diet, preserving normal liver morphology [25].

In short, the results showed that the use of liposomes containing *P. heptaphyllum* extract for 14 days showed an improvement in the functional and inflammatory parameters of the liver in obese animals. However, the suggested treatment was not effective in alleviating general changes related to obesity, such as weight gain, fat, glucose, triglycerides, and inflammation in adipose tissue.

## 5. Final Considerations

The use of nanotechnology to develop liposomes with *P. heptaphyllum* extract sought to improve the effectiveness of the treatment and minimize potential adverse effects. Physicochemical analyses confirmed the quality of the formulations, qualifying the liposomes for application in biological models. In the context of induced obesity in rats, liposomes containing extract, although showing significant benefits in the liver, were not effective in reversing the metabolic, histological, and inflammatory changes associated with obesity. A positive response to treatment was observed, evidenced by the reduction in oxidative stress markers, restoration of metabolic parameters in the liver, and modulation of inflammatory cytokines. However, the treatment did not influence weight gain, glucose levels, triglycerides, or inflammation in adipose tissue, indicating limitations in reversing systemic changes related to obesity. Thus, although promising, liposomes with *P. heptaphyllum* extract proved to be only partially effective given the complexity of obesity manifestations, highlighting the need for more in-depth investigations and complementary strategies.

## Figures and Tables

**Table 1:** Composition of standard feed and high-calorie feed.

Components	Standard Ration	Hypercaloric Ration
Carbohydrates (%)	65.5	45.2
Proteins (casein > 99%) (%)	22	20.9
Lipids (%)	4	24.5
Fibers (%)	4	4
Vitamins * (%)	1	1
Mixture of minerals * (%)	3.5	3.5
Total (%)	100	100
Caloric value (kcal)	3.800	4.849

* Supplemented content of vitamins and minerals per 1000 g of high-calorie feed: iron: 25.2 mg; potassium: 104.8 µg; selenium: 73.1 µg; molybdenum sulfate: 150.0 µg; vitamin B_12_: 34.5 µg; vitamin B_6_: 6 µg; biotin: 0.12 µg; vitamin E: 48.9 IU; vitamin D: 2447.0 IU; and vitamin A: 15291.2 IU.

**Table 2:** Vesicle diameter of equivalent nanocapsules over the 30 days of study.

Method	PhysicaL-Chemical Parameters	LP_BR_	LP_EP_
Laser diffraction	(µm)	220 ± 0.57	287 ± 0.86
	SPAN	1.71 ± 2.41	2.04 ± 0.00
Photon correlation spectroscopy	Z-average (nm)	232 ± 0.00	260 ± 1.70
	PDI	0.210 ± 0.00	0.210 ± 0.01
Electrophoretic mobility	ζ potential (mV)	−16.9 ± 0.37	−20.07 ± 1.10
pH	pH	7.28 ± 0.03	7.35 ± 0.05

Results are presented as the mean ± standard deviation from three batches of the formulation.

**Table 3:** Anthropometric parameters and food consumption.

Parameters	Control (C)	Protium (P)	Obese (O)	Obese + Protium (OP)
Initial body weight (g)	167.00 ± 33.80	185.40 ± 48.66	175.40 ± 38.87	167.60 ± 36.10
Final body weight (g)	346.30 ± 28.36	344.20 ± 42.65	428.70 ± 37.39 *	443.40 ± 44.30 *
Weight gain (g)	12.13 ± 5.46	15.00 ± 2.77	33.13 ± 6.46 *	32.50 ± 8.10 *
Food intake (g/day/rat)	23.35 ± 1.58	23.95 ± 1.63	17.73 ± 2.05 *	17.88 ± 1.74 *
Water intake (mL/day/rat)	36.61 ± 2.28	39.29 ± 2.74	54.89 ± 7.77 *	52.74 ± 7.27 *
Calorie intake (kcal/day/rat)	88.06 ± 5.97	90.32 ± 6.17	157.03 ± 11.12 *	155.20 ± 13.69 *
Epididymal adipose tissue (g)	4.30 ± 0.84	4.81 ± 0.96	8.98 ± 1.27 *	13.24 ± 1.92 *^/#^
Retroperitoneal adipose tissue (g)	5.75 ± 1.37	6.05 ± 2.17	12.88 ± 1.64 *	18.15 ± 2.19 *^/#^
Liver (g)	10.68 ± 1.00	11.01 ± 1.76	13.23 ± 0.62 *	13.59 ± 1.58 *
HSI (hepatosomatic index) (%)	3.13 ± 0.11	3.26 ± 0.39	2.90 ± 0.34	3.11 ± 0.12

Results are presented as mean ± standard deviation. ANOVA (two-way) followed by Tukey’s post-hoc test. * *p* < 0.05 vs. C; # *p* < 0.05 vs. O.

**Table 4:** Plasma biochemical parameters in the treated groups.

Parameters	Control (C)	Protium (P)	Obese (O)	Obese + Protium (OP)
Glucose (mg/dL)	114.40 ± 29.59	136.00 ± 14.86	184.25 ± 9.19 *	177.37 ± 23.31 *
Total proteins (mg/dL)	6.73 ± 0.81	5.56 ± 1.02	5.92 ± 1.20	7.12 ± 0.74
ALT (U/L)	75.50 ± 14.02	62.00 ± 11.22	45.88 ± 6.81 *	40.88 ± 7.84 *
AST (U/L)	48.47 ± 12.68	30.53 ± 7.50 *	29.53 ± 10.81 *	26.88 ± 8.02 *
ALP (U/L)	179.10 ± 26.97	168.80 ± 24.74	115.90 ± 16.106 *	100.40 ± 10.73 *
Creatinine (mg/dL)	1.30 ± 0.11	0.96 ± 0.09 *	0.85 ± 0.17 *	0.71 ± 0.23 *
Amylase (U/L)	623.80 ± 117.30	644.50 ± 78.89	680.40 ± 61.02	713.20 ± 58.05
Lipase (U/L)	25.00 ± 5.90	19.75 ± 2.65	21.63 ± 6.18	25.88 ± 3.52

Results are presented as mean ± standard deviation. ANOVA (two-way) followed by Tukey’s post-hoc test. * *p* < 0.05 vs. C. (ALT: alanine aminotransferase; AST: aspartate aminotransferase; ALP: alkaline phosphatase).

**Table 5:** Plasma biochemical parameters regarding lipid profile.

Parameters	Control (C)	Protium (P)	Obese (O)	Obese + Protium (OP)
Cholesterol (mg/dL)	149.60 ± 17.20	130.00 ± 19.49	135.90 ± 20.67	128.40 ± 14.97
HDL (mg/dL)	44.38 ± 2.82	45.88 ± 6.70	39.63 ± 3.37	39.75 ± 4.95
LDL (mg/dL)	88.35 ± 10.70	73.46 ± 10.07 *	78.28 ± 11.76	73.10 ± 12.11 *
VLDL (mg/dL)	21.43 ± 6.26	13.78 ± 3.18 *	13.20 ± 3.09 *	15.88 ± 2.44 *
Triglycerides (mg/dL)	81.04 ± 9.11	68.88 ± 15.91	72.25 ± 7.24	90.50 ± 10.52 ^#^
TG/HDL (mg/dL)	1.82 ± 0.14	1.50 ± 0.25	1.83 ± 0.25	2.31 ± 0.41 *^/#^

Results are presented as mean ± standard deviation. ANOVA (two-way) followed by Tukey’s post-hoc test. * *p* < 0.05 vs. C; # *p* < 0.05 vs. O. (HDL: high-density lipoprotein; LDL: low-density lipoprotein; VLDL: very low-density lipoprotein; TG/HDL: triglycerides/high-density lipoprotein ratio).

**Table 6:** Oxidative stress parameters analyzed in the adipose tissue of rats submitted to a high-calorie diet in the experimental groups.

Parameters	Control (C)	Protium (P)	Obese (O)	Obese + Protium (OP)
SOD (IU SOD/mg protein)	23.94 ± 3.02	22.58 ± 4.18	27.84 ± 5.95	20.80 ± 2.76 ^#^
CAT (μmol/min/mg protein)	7.30 ± 3.06	5.53 ± 2.28	6.37 ± 2.14	6.00 ± 2.26
GST (µmol GS-DNB/min/mg protein)	77.53 ± 27.06	74.75 ± 34.99	105.40 ± 38.99	78.25 ± 20.63
GSH (µmol GSH/mg protein)	32.91 ± 12.32	50.68 ± 16.42	30.58 ± 12.74	40.94 ± 16.30
ASA (μmol ASA/g tissue)	0.36 ± 0.08	0.33 ± 0.10	0.33 ± 0.06	0.30 ± 0.05
TBARS (nmol MDA/mg protein)	0.60 ± 0.18	0.65 ± 0.29	1.46 ± 0.38 *	1.96 ± 0.59 *
Carbonyl (nmol carbonyl/mg protein)	254.10; 254.90	129.40; 124.98 *	167.90; 141.26	162.80; 116.93
Glucose (μmol/g tissue)	5.29 ± 1.69	2.81 ± 1.96	4.07 ± 2.03	3.71 ± 2.81
Glycogen (μmol/g tissue)	11.85 ± 2.52	8.14 ± 1.86 *	5.04 ± 2.71 *	3.94 ± 0.48 *
Amino acids (mmol/g tissue)	0.0026 ± 0.0009	0.0025 ± 0.0004	0.0018 ± 0.0006	0.0016 ± 0.0004 *
Ammonia (μmol/ammonia g tissue)	0.48 ± 0.26	1.30 ± 0.18 *	1.23 ± 0.11 *	0.36 ± 0.14 ^#^
Lactate (μmol/g tissue)	1.81 ± 0.63	3.17 ± 0.45 *	1.66 ± 0.40	2.03 ± 0.25
Total proteins (mg/mL)	0.46 ± 0.22	0.78 ± 0.37	0.62 ± 0.35	0.58 ± 0.11

Results are presented as mean ± standard deviation. ANOVA (two-way) followed by Tukey’s post-hoc test. For carbonyl analysis, the Kruskall–Walli’s test followed by Dunn’s post-hoc test was used, and values were expressed as median and total range. * *p* < 0.05 vs. C; # *p* < 0.05 vs. O. (SOD: superoxide dismutase; GST: glutathione-S-transferase; ASA: ascorbic acid; TBARS: substances reactive to thiobarbituric acid; carbonyl: carbonylated proteins).

**Table 7:** Oxidative stress parameters analyzed in the liver tissue of rats submitted to a high-calorie diet in the experimental groups.

Parameters	Control (C)	Protium (P)	Obese (O)	Obese + Protium (OP)
SOD (IU SOD/mg protein)	11.06 ± 1.03	12.08 ± 3.85	10.46 ± 2.78	10.50 ± 2.65
Catalase (µmol/min/mg protein)	8.48 ± 1.96	9.42 ± 3.63	9.23 ± 3.01	12.01 ± 1.34
GST (µmol GS-DNB/min/mg protein)	250.10 ± 48.60	298.00 ± 76.00	241.50 ± 74.10	227.50 ± 84.10
GPx (µmol/min/mg protein)	31.60 ± 9.00	39.30 ± 12.30	23.50 ± 7.40	23.40 ± 5.90
GSH (µmol GSH/mg protein)	20.94 ± 5.82	20.26 ± 4.57	21.45 ± 6.73	22.53 ± 7.37
ASA (µmol ASA/g tissue)	3.16 ± 0.65	2.96 ± 0.52	2.81 ± 0.44	3.04 ± 0.44
TBARS (nmol MDA/mg protein)	0.14 ± 0.02	0.22 ± 0.03 *	0.23 ± 0.03 *	0.19 ± 0.04
Carbonyl (nmol carbonyl/mg protein)	15.85 ± 1.04	12.40 ± 2.45	13.31 ± 3.86	14.67 ± 2.63
Glucose (µmol/g tissue)	31.27 ± 10.79	66.01 ± 18.55 *	90.81 ± 25.10 *	62.42 ± 16.62 */#
Glycogen (µmol/g tissue)	1.53 ± 0.24	1.96 ± 0.28 *	1.63 ± 0.32	1.84 ± 0.37
Amino acids (mmol/g tissue)	0.10 ± 0.02	0.08 ± 0.01 *	0.07 ± 0.009 *	0.11 ± 0.01 #
Ammonia (µmol ammonia/g tissue)	1.09 ± 0.33	0.60 ± 0.19 *	0.81 ± 0.27	0.75 ± 0.26
Lactate (µmol/g tissue)	1.66; 1.23	1.82; 1.29	0.85; 0.95 *	1.53; 1.25 #
Total proteins (mg/mL)	6.65 ± 0.21	7.02 ± 2.13	6.58 ± 1.66	7.25 ± 2.46

Results are presented as the mean ± standard deviation. ANOVA (two-way) followed by Tukey’s post-hoc test. For lactate analysis, the Kruskall–Wallis test followed by Dunn’s post-hoc test was used, and the values are expressed as the median and total range. * *p* < 0.05 vs. C; # *p* < 0.05 vs. O. (SOD: superoxide dismutase; GST: glutathione-S-transferase; GPx: glutathione peroxidase; ASA: ascorbic acid; TBARS: substances reactive to thiobarbituric acid; carbonyl: carbonylated proteins).

## Data Availability

The data that support the findings of this study are available from the corresponding author upon reasonable request.
