# Correction: Patias et al. Effect of Liposomal *Protium heptaphyllum* (Alb.) March Extract in the Treatment of Obesity Induced by High-Calorie Diet. *Biology* 2024, *13*, 535

**DOI:** 10.3390/biology13100833

**Published:** 2024-10-17

**Authors:** Naiéle Sartori Patias, Eveline Aparecida Isquierdo Fonseca de Queiroz, Stela Regina Ferrarini, Gisele Facholi Bomfim, Danilo Henrique Aguiar, Adilson Paulo Sinhorin, Alexandre Aymberé Bello, Geovana Vicentini Fazolo da Silva, Larissa Cavalheiro, Valéria Dornelles Gindri Sinhorin

**Affiliations:** 1 Postgraduate Program in Biotechnology and Biodiversity of the Pro Centro-Oeste Network, Federal University of Mato Grosso, Sinop 78550-728, MT, Brazil; nai.sartori@gmail.com (N.S.P.); adilson.sinhorin@ufmt.br (A.P.S.); 2 NUPADS—Health Research and Teaching Support Center, Institute of Health Science, Federal University of Mato Grosso, Sinop 78550-728, MT, Brazil; eveline.queiroz@ufmt.br (E.A.I.F.d.Q.); gisele.bomfim@ufmt.br (G.F.B.); 3 Pharmaceutical Nanotechnology Laboratory, Postgraduate Program in Health Sciences, Federal University of Mato Grosso, Sinop 78550-728, MT, Brazil; stela.ferrarini@ufmt.br; 4 Institute of Natural, Human and Social Sciences, Federal University of Mato Grosso, Sinop 78550-728, MT, Brazil; dha.danilo@gmail.com (D.H.A.); larissacavalheiro@gmail.com (L.C.); 5 Institute of Health Science, Federal University of Mato Grosso, Sinop 78550-728, MT, Brazil; alexandre_aymbere@hotmail.com (A.A.B.); fazolog22@gmail.com (G.V.F.d.S.)

## Error in Table

In the original publication [1], Table 1 was also inserted as Table 2. The correct Table 2 is shown below.

Table 7: The GPx unit has been corrected from nmol/min/mg protein to µmol/min/mg protein. In addition, we have corrected the data for ASA in the OP group, where the value has been adjusted from 3.045 to 3.04. The corrected Table 7 is also attached.

The authors state that the scientific conclusions are unaffected. This correction was approved by the Academic Editor. The original publication has also been updated.

**Table 2:** Vesicle diameter of equivalent nanocapsules over the 30 days of study.

Method	PhysicaL-Chemical Parameters	LP_BR_	LP_EP_
Laser diffraction	(µm)	220 ± 0.57	287 ± 0.86
	SPAN	1.71 ± 2.41	2.04 ± 0.00
Photon correlation spectroscopy	Z-average (nm)	232 ± 0.00	260 ± 1.70
	PDI	0.210 ± 0.00	0.210 ± 0.01
Electrophoretic mobility	ζ potential (mV)	−16.9 ± 0.37	−20.07 ± 1.10
pH	pH	7.28 ± 0.03	7.35 ± 0.05

Results are presented as the mean ± standard deviation from three batches of the formulation.

**Table 7:** Oxidative stress parameters analyzed in the liver tissue of rats submitted to a high-calorie diet in the experimental groups.

Parameters	Control (C)	Protium (P)	Obese (O)	Obese + Protium (OP)
SOD (IU SOD/mg protein)	11.06 ± 1.03	12.08 ± 3.85	10.46 ± 2.78	10.50 ± 2.65
Catalase (µmol/min/mg protein)	8.48 ± 1.96	9.42 ± 3.63	9.23 ± 3.01	12.01 ± 1.34
GST (µmol GS-DNB/min/mg protein)	250.10 ± 48.60	298.00 ± 76.00	241.50 ± 74.10	227.50 ± 84.10
GPx (µmol/min/mg protein)	31.60 ± 9.00	39.30 ± 12.30	23.50 ± 7.40	23.40 ± 5.90
GSH (µmol GSH/mg protein)	20.94 ± 5.82	20.26 ± 4.57	21.45 ± 6.73	22.53 ± 7.37
ASA (µmol ASA/g tissue)	3.16 ± 0.65	2.96 ± 0.52	2.81 ± 0.44	3.04 ± 0.44
TBARS (nmol MDA/mg protein)	0.14 ± 0.02	0.22 ± 0.03 *	0.23 ± 0.03 *	0.19 ± 0.04
Carbonyl (nmol carbonyl/mg protein)	15.85 ± 1.04	12.40 ± 2.45	13.31 ± 3.86	14.67 ± 2.63
Glucose (µmol/g tissue)	31.27 ± 10.79	66.01 ± 18.55 *	90.81 ± 25.10 *	62.42 ± 16.62 */#
Glycogen (µmol/g tissue)	1.53 ± 0.24	1.96 ± 0.28 *	1.63 ± 0.32	1.84 ± 0.37
Amino acids (mmol/g tissue)	0.10 ± 0.02	0.08 ± 0.01 *	0.07 ± 0.009 *	0.11 ± 0.01 #
Ammonia (µmol ammonia/g tissue)	1.09 ± 0.33	0.60 ± 0.19 *	0.81 ± 0.27	0.75 ± 0.26
Lactate (µmol/g tissue)	1.66; 1.23	1.82; 1.29	0.85; 0.95 *	1.53; 1.25 #
Total proteins (mg/mL)	6.65 ± 0.21	7.02 ± 2.13	6.58 ± 1.66	7.25 ± 2.46

Results are presented as the mean ± standard deviation. ANOVA (two-way) followed by Tukey’s post-hoc test. For lactate analysis, the Kruskall–Wallis test followed by Dunn’s post-hoc test was used, and the values are expressed as the median and total range. * *p* < 0.05 vs. C; # *p* < 0.05 vs. O. (SOD: superoxide dismutase; GST: glutathione-S-transferase; GPx: glutathione peroxidase; ASA: ascorbic acid; TBARS: substances reactive to thiobarbituric acid; carbonyl: carbonylated proteins).
